# Supplementary material for: The undulating tripod gait as a model of the locomotion of walking fish
Source: Nat Commun. 2026 Jun 2;17:4596. doi: 10.1038/s41467-026-73111-2 (PMC13230637; doi:10.1038/s41467-026-73111-2)
Supplement: Supplementary file 1 — Supplementary Information [file 41467_2026_73111_MOESM1_ESM.pdf]

Supplementary Material

Supplementary Figures

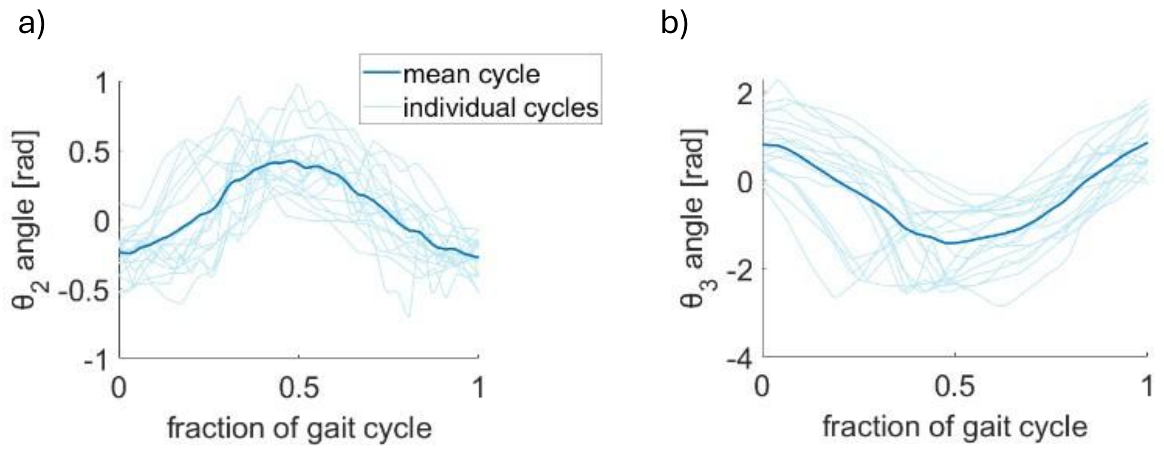

Supplementary Figure 1: Tracking body joint positions individual gait cycles of catfish. a) Angle of the head θ_2 and b) angle of the tail θ_3. Individual tracked cycles are in light colors and the mean of the tracked cycles is plotted as the dark trace, where n=22.

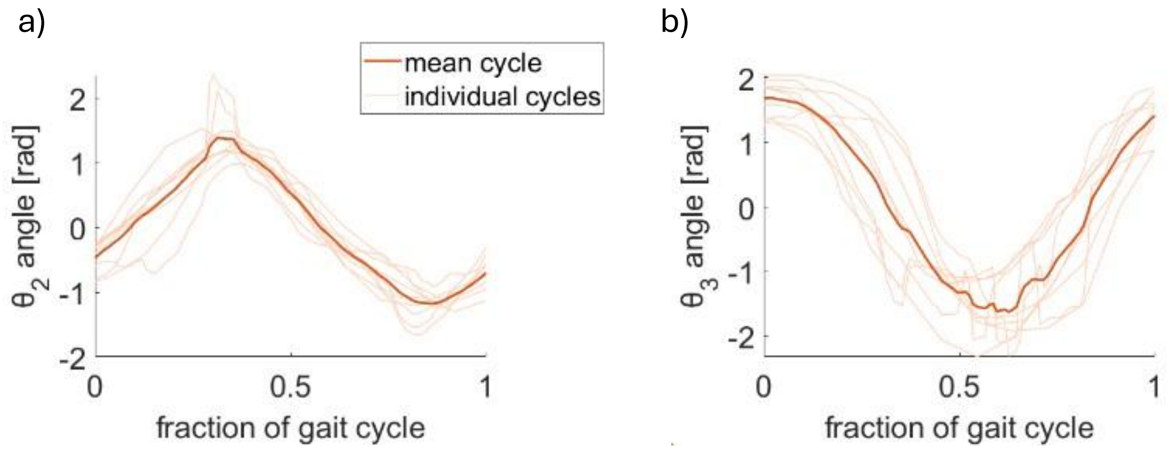

Supplementary Figure 2: Tracking body joint positions individual gait cycles of bichirs. a) Angle of the head θ_2 and b) angle of the tail θ_3. Individual tracked cycles are in light colors and the mean of the tracked cycles is plotted as the dark trace, where n=9.

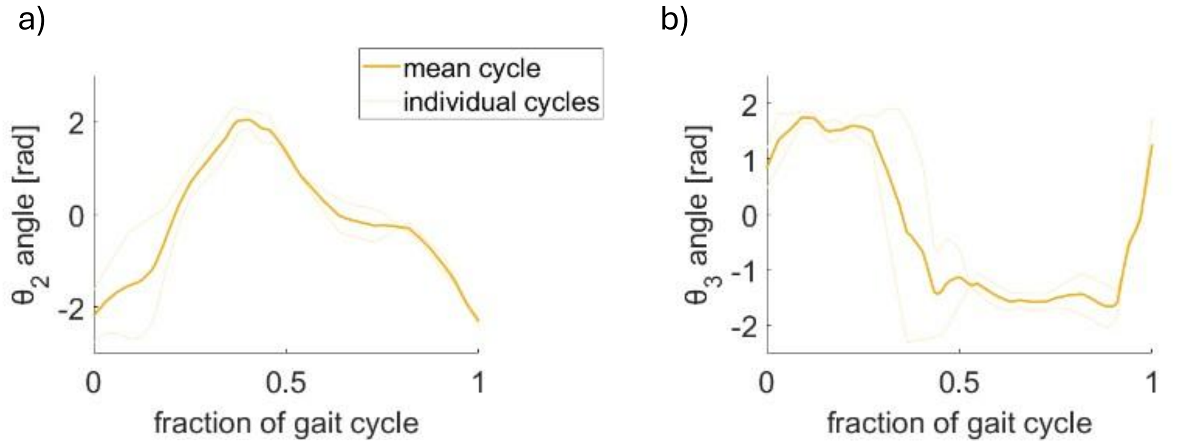

Supplementary Figure 3: Tracking body joint positions individual gait cycles of lungfish. a) Angle of the head θ_2 and b) angle of the tail θ_3. Individual tracked cycles are in light colors and the mean of the tracked cycles is plotted as the dark trace, where n=2.

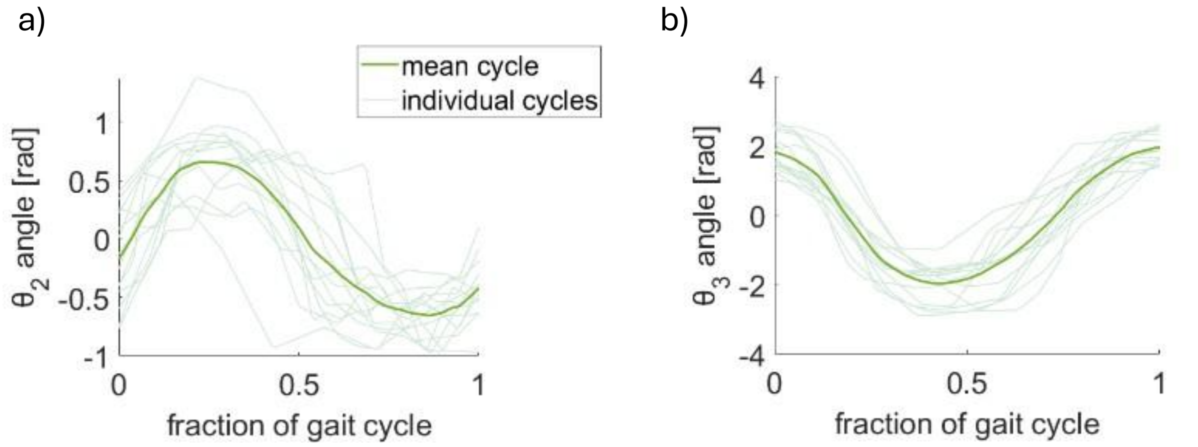

Supplementary Figure 4: Tracking body joint positions for individual gait cycles of snakeheads. a) Angle of the head θ_2 and b) angle of the tail θ_3. Individual tracked cycles are in light colors and the mean of the tracked cycles is plotted as the dark trace, where n=14.

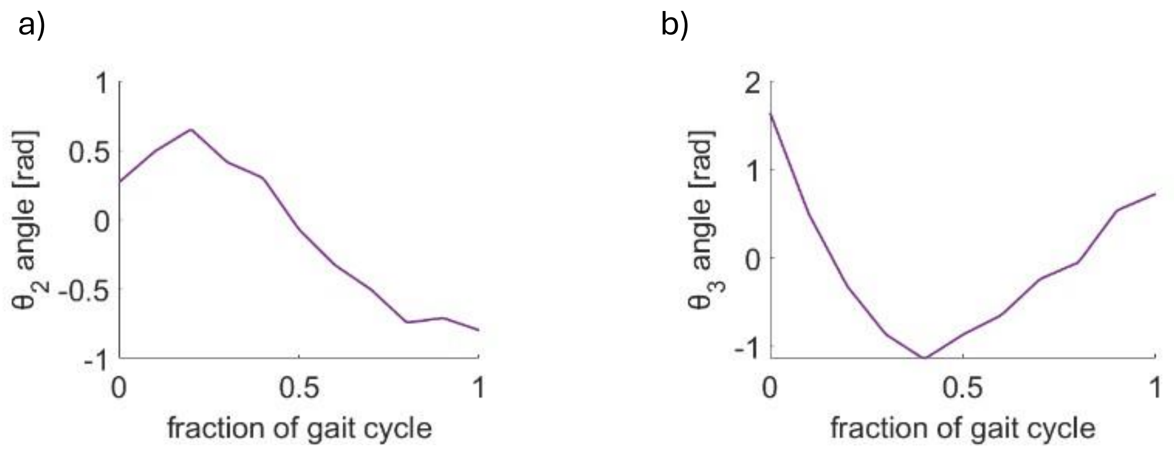

Supplementary Figure 5: Tracking body joint positions for individual gait cycles of a sculpin. a) Angle of the head θ_2 and b) angle of the tail θ_3. Individual tracked cycles are in light colors and the mean of the tracked cycles is plotted as the dark trace, where n=1.

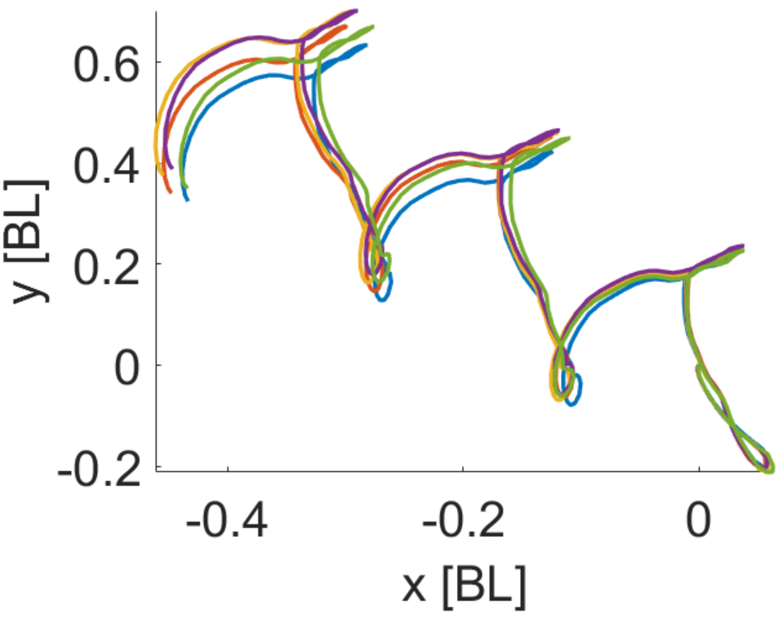

Supplementary Figure 6: X-Y trajectory of the head of the physical robot (n = 5 trials, 3 gait cycles each) with the fastest gait (f = 0.5 Hz, A_2 = 1.5, A_3 = 2.1, $\phi_2 = \pi/2$, $\phi_3 = \pi$).

Supplementary Tables

Supplementary Table 1: Speed of the robot-scale simulation with frozen joint configurations

Scenario	Fish scale (2.5 Hz)	Robot scale (0.5 Hz)
Base gait $\theta_2 = 1.5, \theta_3 = 2.1$	0.334 BL/s	0.261 BL/s
Head amplitude = 0 $\theta_2 = 0, \theta_3 = 2.1$	-0.049 BL/s	-0.024 BL/s
Tail amplitude = 0 $\theta_2 = 1.5, \theta_3 = 0$	0.083 BL/s	0.016 BL/s

Supplementary Table 2: Parameters for simulated ground contact in all simulations

(smooth spring-damper normal force, smooth stick-slip friction)

**Ground stiffness**	1e6 N/m
**Ground damping**	1e5 N/(m/s)
**Transition region width**	1e-4 m
**Coefficient of static friction**	0.5
**Coefficient of dynamic friction**	0.3
**Critical velocity**	1e-3 m/s

Supplementary Table 3: Parameters of physical robot

**Mass of head segment**	157 g
**Mass of middle segment**	280 g
**Mass of tail segment**	63 g
**Length of head segment**	68 mm
**Length of middle segment**	134 mm
**Length of tail segment**	170 mm

Supplementary Table 4: Per-cycle sinusoidal fitting parameters for the phase of *P. senegalus*

Parameter	Mean value	Standard deviation
Amplitude of head joint A_2	-1.17	0.20
Amplitude of tail joint A_3	1.73	0.29
Phase of head joint ϕ_2	2.53	0.22
Phase of tail joint ϕ_2	1.12	0.38
Difference in phases ($\phi_2 - \phi_3$)	1.40	0.31
Offset constant of head joint C_2	0.0032	0.10
Offset constant of tail joint C_3	0.0015	0.24

These parameters are fit to the equation $\theta_n = A_n \sin(2\pi t + \phi_n) + C_n$. We do not fit a frequency parameter as the data has already been normalized per-cycle, but we do fit an amplitude offset parameter.
