# Supplementary material for: The undulating tripod gait as a model of the locomotion of walking fish
Source: Nat Commun. 2026 Jun 2;17:4596. doi: 10.1038/s41467-026-73111-2 (PMC13230637; doi:10.1038/s41467-026-73111-2)
Supplement: Supplementary file 2 — Descriptions of Additional Supplementary Files [file 41467_2026_73111_MOESM2_ESM.pdf]

Description of Additional Supplementary Files

Supplementary Movie 1: Walking motion of *Polypterus senegalus*

Supplementary Movie 2: Effect of head amplitude on simulated robot locomotion

Supplementary Movie 3: Effect of tail amplitude on simulated robot locomotion

Supplementary Movie 4: Effect of phase on the direction of simulated robot locomotion

Supplementary Movie 5: Effect of body proportions on simulated robot locomotion

Supplementary Movie 6: Locomotion of the Physical Robot

Supplementary Movie 7: Effect of deactivating individual body joints on simulated robot locomotion
